# An Accurate Measurement Method for Azimuth Pointing of Spaceborne Synthetic Aperture Radar Antenna Beams Based on Ground Receiver

**DOI:** 10.3390/s18082626

**Published:** 2018-08-10

**Authors:** Weibin Liang, Zengzeng Jia, Lihong Kang, Jun Hong, Bin Lei, Qingjun Zhang, Qi Chen

**Affiliations:** 1 Key Laboratory of Technology in Geo-Spatial Information Processing and Application Systems, Chinese Academy of Sciences, Beijing 100190, China; 13051561667@163.com (Z.J.); jhong@mail.ie.ac.cn (J.H.); leibin@mail.ie.ac.cn (B.L.); 2 Institute of Electronics, Chinese Academy of Sciences, Beijing 100190, China; 3 School of Artificial Intelligence, University of Chinese Academy of Sciences, Beijing 100190, China; 4 Beijing institute of Remote Sensing Information, Beijing 100192, China; gregrs@126.com; 5 National Key Laboratory of Microwave Imaging Technology, Beijing 100190, China; 6 Beijing Institute of Space System Engineering, China Academy of Space Technology, Beijing 100086, China; ztzhangqj@163.com; 7 China Centre for Resource Satellite Data and Application, Beijing 100094, China; chenq_cn@163.com

**Keywords:** Gaofen-3, SAR, calibration, antenna beam pointing, Image radiation correction

## Abstract

The paper proposes a new method for measuring the azimuth pointing of spaceborne synthetic aperture radar (SAR) antenna beams based on the ground receiver, which can receive and record complex sampling data of the pulse signals transmitted from the spaceborne SAR. The center of the antenna pattern is extracted from the complex sampling data amplitude envelope to obtain the time when the beam main lobe center irradiates the ground receiver, and the range migration information is extracted from the complex sampling data to obtain the time when the satellite is over the top of the ground receiver. The results of Chinese civilian remote sensing GaoFen-3 SAR satellite experiment data processing show that the measurement accuracy of this method is better than 0.002°, which can be applied to the accurate measurement of azimuth pointing of various low Earth orbit (LEO) SAR antenna beams.

## 1. Introduction

Spaceborne synthetic aperture radar (SAR) antenna pattern modulation is the main error source affecting the SAR image radiation precision. Therefore, antenna pattern measurement is a major aspect of spaceborne SAR radiation calibration [1]. The spaceborne SAR antenna pattern is usually tested at the ground stage, however, it is impossible to fully achieve far-field conditions or to completely simulate the space environment on the ground. It is necessary to perform on-orbit testing of the antenna pattern in practical applications. The main factor that determines the accuracy of the antenna pattern correction is the measurement accuracy of the antenna pattern shape and the alignment accuracy between the SAR data and the antenna pattern. Therefore, the on-orbit measurement of the antenna pattern of the spaceborne SAR is divided into two aspects: the shape of the antenna pattern measurement and antenna beam pointing measurement.

Since the launch of SEASAT, the first spaceborne SAR system in the United States in 1978, various spacefaring nations have launched their own SAR satellites, such as SIR-A, SIR-B, ERS-1, ERS-2, JERS-1, and RADARSAT-1. During the 21st century, SAR satellites have been launched more frequently, including ENVISAT, ALOS, SAR-Lupe, COSMO-SkyMed, TerraSAR-X, Radarsat-2, TanDEM-X and so on [2]. In order to quantify the SAR data, these spaceborne SARs have been tested on the antenna pattern on-orbit. Scientists have developed a complete series of on-orbit test methods to test the shape of antenna patterns, such as the standard reflector method [3], ground receiver method [4,5], ground transmitter method [6,7], and tropical rainforest uniform target method [8,9,10,11,12,13,14]. As the number of beams increased, TerraSAR-X developed the antenna pattern measurement and verification method based on a mathematical model [15,16]. As For the antenna beam pointing measurement, little mention was made in the early days of spaceborne SAR. The DLR designed and implemented a calibration method for TerraSAR-X to measure the antenna beam pointing via transmitting a special kind of antenna beam with a notch pattern, and the accuracy in range direction was better than 0.008° [16]. The beam pointing in the azimuth direction could be determined likewise by notch patterns now operated in the azimuth direction and measured by deployed ground receivers, with accuracy better than the required 0.002° [17].

However, the beam pointing measurement method via the notch beam requires a specifically designed operating mode, and this makes most of the antenna energy unable to be emitted, as it will lead to the antenna overheating. There is a risk of burning down the antenna device, which is unacceptable. In this paper, we propose a high-precision measurement technique for the azimuth direction of the SAR antenna beam based on the ground receivers, which solves the above problems while achieving an accuracy of 0.002°. Since no special adjustments are required for satellites and payloads, this method is more widely applicable. This paper firstly introduces the principle of the new measurement method in Section 2, and then describes the specific algorithm in Section 3. In Section 4, we analyze the accuracy of our method. With the experiment data of the GaoFen-3 SAR satellite, the antenna beam azimuth direction measurement results are given in Section 5. Finally, In Section 6, we illustrate the effectiveness of the method.

## 2. Methodology

### 2.1. Knowledge and Definition

**Figure 1:**
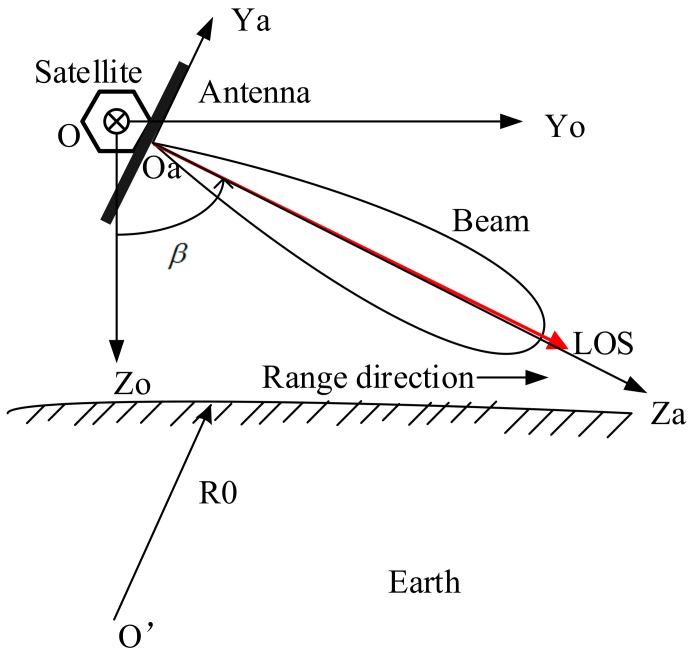
Antenna beam pointing diagram in the orbital coordinate system.

Spaceborne SAR antenna beam pointing can be expressed by the line of sight (LOS) of the antenna in the satellite orbital coordinate system $OX_{o}Y_{o}Z_{o}$, as shown in the Figure 1. The satellite speed direction points to the inside of the paper, which is denoted as the $X_{o}$ direction. $Z_{o}$ direction is determined by the center of gravity of the satellite and the center of the earth and the $Y_{o}$ direction is determined by the right-hand rule. $V_{LOS}$ is the unit vector of LOS whose direction is that of the centerline of the antenna pattern, along which the antenna pattern has the strongest energy. In general, the antenna pattern is symmetrical about the centerline, for the convenience of description, this paper discusses this situation. The coordinates of the vector $V_{LOS}$ in the orbital coordinate system are:

(1) $$S_{o-LOS}={\left[x_{o},y_{o},z_{o}\right]}^{T}$$

The beam pointing squint angle is:

(2) $$\alpha =\frac{\pi }{2}-\cos^{-1}(V_{LOS}\cdot X_{o})$$

The beam pointing look angle is:

(3) $$\beta =\frac{\pi }{2}-\cos^{-1}(V_{LOS}\cdot Y_{o})$$

**Figure 2:**
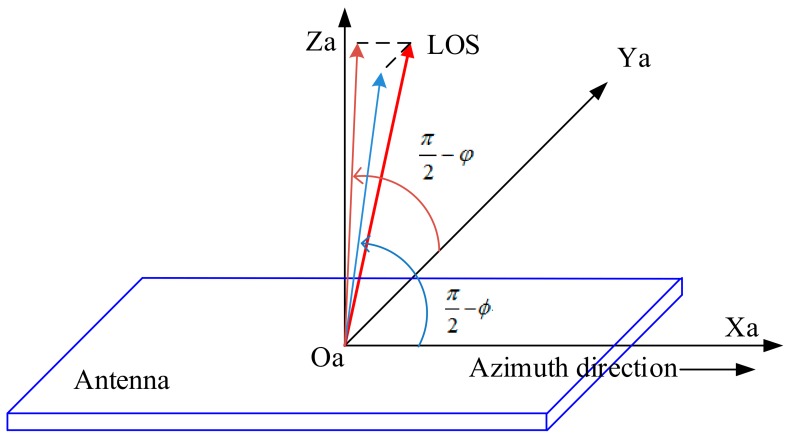
Azimuth and elevation angle of the antenna beam.

The squint angle α and look angle β represent the direction of the beam itself in space, but sometimes it needs to know the direction of the beam in the antenna coordinate system $O_{a}X_{a}Y_{a}Z_{a}$, i.e., the beam direction represented by the antenna beam azimuth and elevation angle, as shown in Figure 2. In the antenna coordinate system, $X_{a}$ points to the direction of the length of the antenna, $Y_{a}$ points to the direction of the height of the antenna, and $Z_{a}$ points to the direction of the normal of the antenna. The coordinates of the vector $V_{LOS}$ in the antenna coordinate system are:

(4) $$S_{a-LOS}={\left[x_{a},y_{a},z_{a}\right]}^{T}$$

The beam pointing azimuth angle is:

(5) $$\phi =\frac{\pi }{2}-\cos^{-1}(V_{LOS}\cdot X_{a})$$

The beam pointing elevation angle is:

(6) $$\varphi =\frac{\pi }{2}-\cos^{-1}(V_{LOS}\cdot Y_{a})$$

The following formula gives the conversion relation of the description of the vector $V_{LOS}$ from the orbital coordinate system to the antenna coordinate system:

(7) $$S_{a-LOS}=C_{star2antenna}C_{orbit2star}S_{o-LOS}$$

where, $C_{orbit2star}$ is the transition matrix from the orbital coordinate system to the satellite body coordinate system measured by the satellite attitude sensors, $C_{star2antenna}$ is the transition matrix from the satellite body coordinate system to the antenna coordinate system measured from the ground. In Appendix A, we give the specific expressions of these two matrices.

The beam pointing error can be divided into three parts: First, the orbit error, whose contribution is relatively small. Second, the attitude control error. The attitude control system is a closed-loop control system with the satellite attitude sensors as the measurement component because the satellite attitude sensor’s accuracy is very high and the contribution of this part of the error is also relatively small. Third, the antenna error, which is the greatest error, including the relative installation error between the satellite attitude sensors and the antenna, and the beam pointing error caused by the antenna’s own electromechanical and thermal factors. The antenna is associated with the satellite attitude sensors through the installation matrix $C_{antenna2star}$. Although the installation matrix is accurately measured on the ground, as a result of the launching vibration environment and the stress release in orbit, the actual installation relationship will change and cannot be directly measured. Antenna error is the most important error, which can only be resolved by on-orbit calibration and attitude closed-loop control. However, it is difficult to measure the antenna’s own beam pointing error directly on orbit. Generally, we measure the value of the vector $V_{LOS}$ in the orbital coordinate system; i.e., $S_{o-LOS}$, and then obtain $S_{a-LOS}$ by (7).

For spaceborne SAR, the imaging performance is affected by the squint and look angles of the antenna beam in the orbital coordinate system, and the on-orbit measurement also directly measures these two angles, and then obtains the nominal azimuth angle and elevation angle according to the conversion relationship (7). If the squint and look angles do not meet the imaging requirements, the beam pointing is adjusted by adjusting the satellite attitude angles.

### 2.2. Measuring Principle

For LEO spaceborne SAR with phased array antennas, when the satellite is in orbit, ignoring yawing guidance, the direction of the antenna $X_{a}$ is generally consistent with the direction of the satellite speed. As shown in Figure 1, the antenna beam squint angle and the azimuth angle are equal. In fact, when the satellite is in orbit, what the ground can directly feel is the squint angle of the antenna beam, and this angle directly affects the azimuth imaging performance. Therefore, the so-called antenna beam azimuth pointing measurement on-orbit is the measurement of this squint angle.

This paper focuses on the method for measuring the squint angle of the antenna beam in the orbital coordinate system. Furthermore, the beam pointing test is performed while the SAR is in the side looking mode, with the beam squint angle being close to zero.

Assuming that the spaceborne SAR is orbiting, a ground receiver is placed near the center of the ground imaging area to receive the pulse signal transmitted by the spaceborne SAR. The signal is sampled and recorded at a certain frequency which meets the sampling theorem. We define the closest relative position of the spaceborne SAR and ground receiver as the position where the satellite is right over-the-top of the receiver (this moment is denoted as the satellite’s ceiling time). At this position, the connection line between the satellite and the ground receiver is approximately perpendicular to the satellite’s flight trajectory.

**Figure 3:**
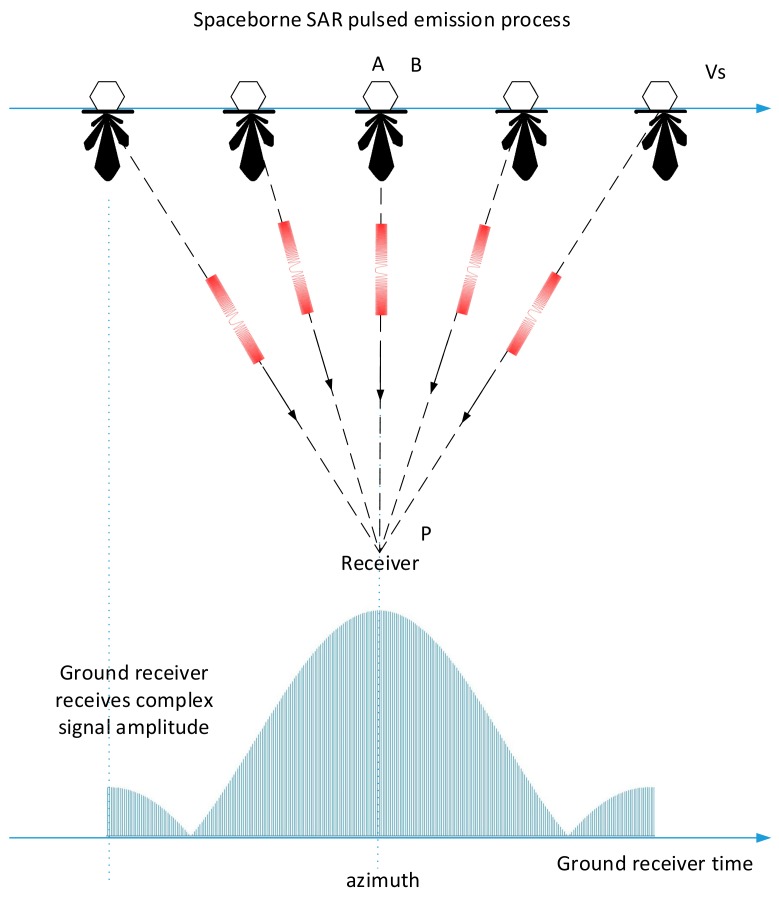
The situation where the squint angle of the antenna beam is 0.

When the center line of the spaceborne SAR’s antenna azimuth is aligned with the ground receiver, this moment is defined as the beam alignment time. As shown in the Figure 3, at the beam alignment time (the corresponding position is point B in Figure 3), if the satellite is right over-the-top of the ground receiver (the corresponding position is point A in Figure 3), the azimuth pointing of the antenna is perpendicular to the satellite flight trajectory, which indicates that the squint angle is 0. After the corrections of range power attenuation and the ground receiver antenna pattern’s weighting, the amplitude envelope of the SAR’s pulse signal received by the ground receiver is symmetrical about point A (and point B).

**Figure 4:**
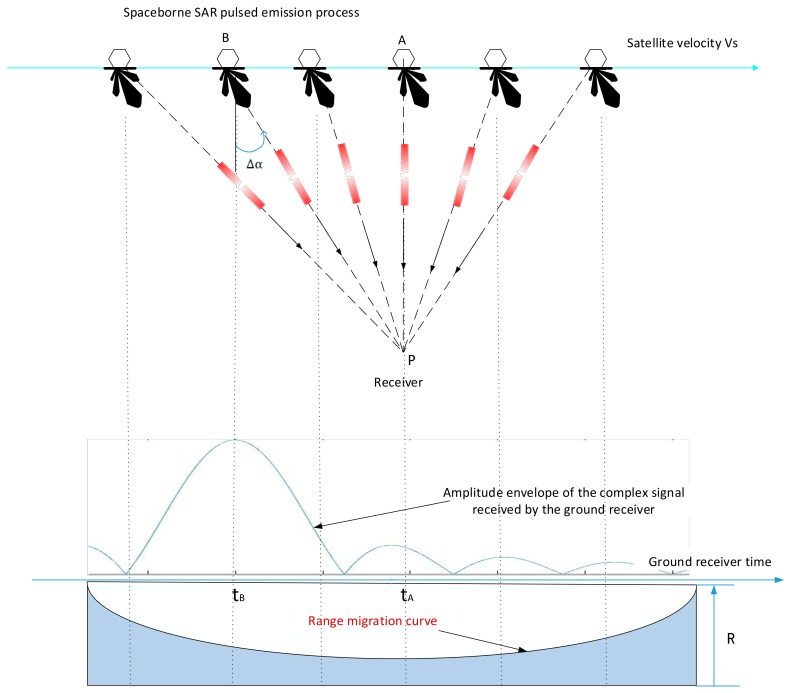
The situation where the squint angle of the antenna beam is not 0. R denotes the distance between the satellite and the ground receiver.

If the azimuth of the SAR’s antenna beam deviates from the plane of the vertical satellite flight trajectory with an angle Δα, that is, the squint angle is Δα, as shown Figure 4, then when the satellite is over-the-top of the ground receiver, corresponding to point A, after the corrections of range power attenuation and the ground receiver antenna pattern’s weighting, the amplitude envelope of complex sampling data received by the ground receiver is asymmetrical about this point. When the beam is aligned with the ground receiver at point B, the amplitude envelope is symmetrical about this point. Ignoring the orbital curvature of the satellite, according to the geometric relationship, ∠BPA is equal to Δα, which is the azimuth pointing of the antenna beam.

In order to obtain ∠BPA. which is Δα accurately, it is necessary to know the satellite orbit, the position of the ground receiver, the length of the line segment AB. Δα is as (8):

(8) $$\Delta \alpha =tg^{-1}[\frac{(t_{A}-t_{B})V_{S}}{R_{0}}]$$

where $R_{0}$ is the distance between the satellite and the ground receiver at the satellite’s ceiling time, $V_{S}$ is the velocity of the satellite, $t_{A}$ is the satellite’s ceiling time, $t_{B}$ is the beam alignment time.

The satellite orbital ephemeris data, the position of the ground receiver, and the velocity of the satellite are all relatively easy to obtain. The key to this method lies in determining $t_{A}$ and $t_{B}$.

### 2.3. Extracting $t_{A}$

According to the working mechanism of spaceborne SAR, while the satellite is in work, the chirp signal is transmitted at a fixed pulse repetition frequency (PRF) while the ground receiver receives the SAR chirp signal. Since the distance between the satellite and the ground receiver is constantly changing, there exists range migration effect. Pulse signals that are transmitted at equal intervals will not be equally spaced when received at the ground receiver. The changes in the interval of the received pulse signal reflect the changes in the spatial relationship of the satellite and the ground receiver. Figure 4 shows this process. It can be seen that at the time $t_{A}$ the satellite is right over-the-top of the ground receiver, corresponding to the nearest position of the satellite, about which the range migration curve is symmetrical. According to these characteristics the time $t_{A}$ can be extracted.

### 2.4. Extracting $t_{B}$

For operability, we ignore the influence of the antenna side lobes. Considering the mechanism of SAR azimuth imaging, it is very reasonable that the azimuth beam center is defined as the center of the main lobe of the azimuth antenna pattern. When the spaceborne SAR is operating, chirp signals are transmitted at a fixed frequency. After the correction of range power attenuation and the weighting of the ground receiver antenna pattern, the envelope of the SAR pulse signals recorded by the ground receiver reflect the azimuth energy distribution of the antenna beam, which could be used to measure the azimuth emission pattern. According to the definition of the beam azimuth direction, we can obtain the $t_{B}$ through calculating the azimuth antenna pattern center.

## 3. Data Processing Algorithm

Since the range migration curve is very flat, for a spaceborne SAR with a resolution of the order of meters, the range migration in the synthetic aperture time is not obvious, which makes it difficult to accurately extract the center position of the range migration curve. The center position of the main lobe of the spaceborne SAR azimuth antenna pattern is also very flat, making it hard to extract the center position. In addition, generally, ground receivers only record signals near the center location. All of these make it a challenge to extract $t_{A}$ and $t_{B}$ accurately, which requires specifically designed algorithms.

### 3.1. Algorithm of Extracting $t_{A}$

**Figure 5:**
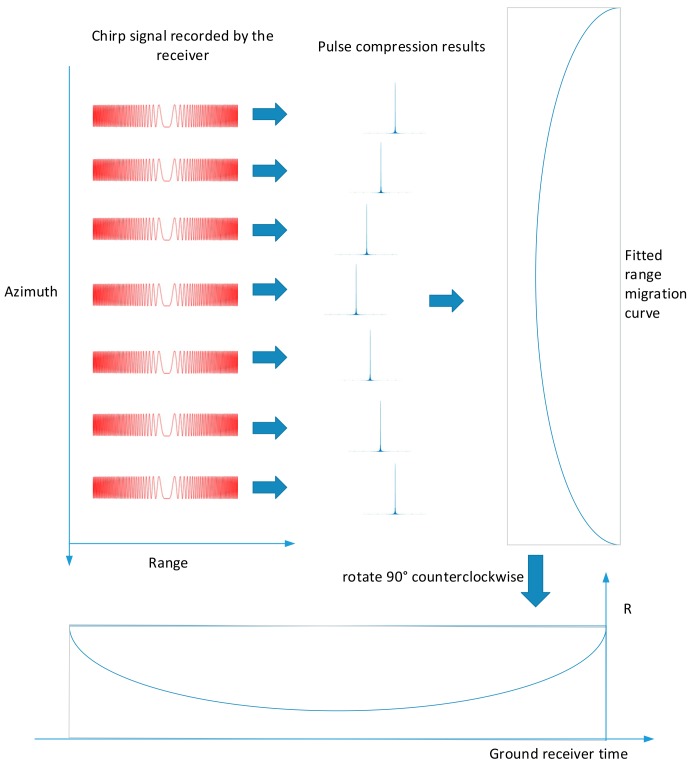
Range migration information extraction process.

Since the ground receiver completely records the phase information of the pulse signal transmitted from the spaceborne SAR, the effect of range migration can be amplified by interpolation, which improves the accuracy of $t_{A}$ extraction. Figure 5 shows the process, and the process is as follows:

(1) Intercept the complex sampling data of the pulse signal received by the ground receiver according to the pulse repetition time (PRT), and remove the same amount of redundant noise data in each cycle;
(2) Perform interpolation to obtain some continuously changing pulse compression signals;
(3) Calculate the position of each pulse peak in range direction to get the range migration curve;
(4) According to the range migration curve, calculate the azimuth time corresponding to the closest pulse peak from the satellite to the ground receiver, which is $t_{A}$.

#### 3.1.1. Pulse Compression

Pulse compression is performed to compress the wide pulse into a narrow one, making the output signal appear at the target’s range cell, which increases the signal to noise ratio.

The SAR system is linear and the resulting image after pulse compression is represented by the sinc function.3.1.2. Interpolation

The signal after the pulse compression is discrete, with unsmooth transitions and some steep edges at the peak. Interpolation is used for smoothing in order to accurately find the peak position. The interpolation process is as follows:

(1) Fourier transform the compressed signal to the frequency domain;
(2) Perform zero-added (power of 2) in the noise region outside the signal bandwidth;
(3) Perform inverse Fourier transform to complete the time-domain interpolation so that the time-domain signal is smooth and continuous.

Another function of the interpolation is to make the characteristic of range migration more obvious. The range migration is approximately 1–2 cells, which is relatively small. Through interpolation, the migration is magnified, which might reach the interpolation multiplier, making the measurement of the $t_{A}$ more accurate.

#### 3.1.3. Range Migration Curve Extraction

After interpolation, each pulse is arranged in the manner shown in Figure 5. The range migration curve extraction process is as follows:

(1) Find the maximum value of each pulse.
(2) Record the corresponding azimuth and range position.
(3) Obtain the range migration curve.

#### 3.1.4. Extracting $t_{A}$

After Knowing the range migration curve R(t,τ), we could construct the fitted function $R_{f}(t,\tau )$. The process for extracting $t_{A}$ is as follows:

(1) Find N points far away from the minimum of the curve at one side, and note the abscissa (Pulse number position) as well as the ordinate values (Interpolated range cell position).
(2) Find other N points at the other side, whose ordinates are equal to the first N points, and note their abscissas.
(3) Obtain the satellite’s ceiling time $t_{A}$ by calculating the mean value of the abscissas of the 2N points.

The larger the N is, the more accurate the obtained $t_{A}$ is.

### 3.2. Algorithm of Extracting $t_{B}$

Due to the gradual change in the distance between the satellite and the ground receiver, the amplitude envelope of the SAR pulse signal recorded by the ground receiver is slightly different from the antenna pattern. In the area near the main lobe peak of the antenna beam, the change of the distance is very small (only a few range cells in the entire synthetic aperture time), whose influence could be ignored. In addition, the ground receiver’s own antenna pattern also modulates the received SAR pulse signal, but since the ground receiver beamwidth is several tens of times greater the SAR beamwidth (generally 50 times), in the case of a near side looking, the flattest portion of the main lobe of the receiver antenna is used to measure the SAR antenna pattern, which means the effect of the modulation is very small and could be ignored. Therefore, we can calculate the energy center of the main lobe with the received data envelope instead of the antenna azimuth pattern.

The pulse compression peak of the range migration curve obtained above corresponds to the amplitude envelope of the data received by the ground receiver, which also reflects the azimuth pattern of the antenna of the spaceborne SAR. Record the pulse azimuth position and pulse compression peak of the range migration curve, and obtain the azimuth pattern function P(t), then construct the fitted function $P_{f}(t)$. Using the antenna main lobe symmetry property, we could obtain the abscissa $t_{0}$ of the antenna main lobe’s maximum value with the same method of obtaining $t_{A}$.

**Figure 6:**
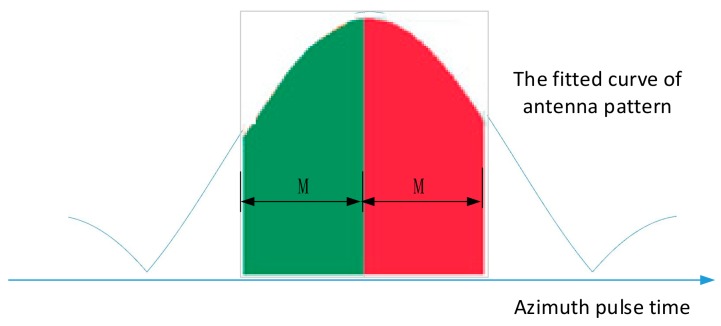
Antenna pattern energy center solution.

The symmetry of the energy of the main lobe could be used to verify and correct $t_{0}$. The method is formula (9), find a series of $t_{i}$ near $t_{0}$, calculate the integral of the left M points, and then calculate the integral of the right M points, then the left integral subtract the right integral and find the modulo value $\Delta S(t_{i},M)$. The best estimation of the center of the antenna beam corresponds to the smallest modulus $\Delta S(t_{i},M)$, so as to obtain $t_{B}$. In the main lobe range, and the data allowable conditions, the larger the value of M, the more accurate the center of antenna pattern energy obtained. The process is shown in Figure 6.

(9) $$\Delta S(t_{i},M)=\left|\int_{t_{i}-M}^{t_{i}}P_{f}(t)dt-\int_{t_{i}}^{t_{i}+M}P_{f}(t)dt\right|$$

## 4. Measurement Accuracy Analysis

According to Equation (8), the antenna beam azimuth pointing measurement error includes the measurement error $\Delta t_{A}$ at the satellite’s ceiling time, the measurement error $\Delta t_{B}$ at the beam alignment time, satellite velocity error $\Delta V_{S}$, and space-to-earth range error $\Delta R_{0}$. The satellite speed measurement error is within 1m/s, the precision track data can reach 0.1 m/s [18,19,20]; after the slant range calibration, the accuracy of the slant range can reach 1 m [21,22]. Since the orbital altitude of LEO SAR satellites is generally in the range of 500 km to 800 km [23], and the speed is approximately 7500 m/s [24], the ratio of satellite speed measurement error and the ratio of slant range measurement error is very small, and the resulting error can be ignored.

For the SAR transmit antenna pattern, the main lobe is relatively wide, and it is difficult to accurately determine the peak position. The energy center of the beam main lobe can be determined by the algorithm in Section 3, when the received data is complete and the value of N is large, and the measurement error theoretically does not exceed 1 PRT. However, due to the receiver’s measurement error, improper recording start time, or insufficient data recording time, the data processing cannot achieve the desired effect, which causes the measured pulse position $t_{B}^{\prime }$ to be inconsistent with the theoretical position $t_{B}$, and introduces measurement error. This measurement error is equal to the difference between the extreme position of the measured data and the extreme position of the theoretical data. Since the theoretical data is not available, we use the fitting data $P_{f}(t)$ instead of the theoretical data to obtain an estimate of the difference between the two. To synthesize the error of two parts, the error of measuring the beam center position is:

(10) $$\delta t_{B}=\left|t_{B}^{\prime }-t_{B}\right|+1$$

Range migration is also a very slow process. For low-resolution spaceborne SAR, only one or two range gates may be used in the synthetic aperture time, through the method of Section 3, the range pulse compression data is interpolated (generally 32 multiples are enough), and the range migration gate’s amount can be enlarged to interpolation multiples, and achieve accurate positioning, and the measurement error theoretically does not exceed 1 PRT. However, due to various reasons, the measured data is not ideal, which causes the measured pulse position $t_{A}^{\prime }$ to be inconsistent with the theoretical position $t_{A}$ and introduces measurement error. Similarly, this measurement error is equal to the difference between the actual measurement data extreme position and the theoretical data extreme value position. Since the theoretical data is not available, here we use the fitting data $R_{f}(t,\tau )$ instead of the theoretical data to obtain an estimate of the difference between the two. To synthesize the error of two parts, the error $\delta t_{A}$ of measuring the center position of the range migration curve is:

(11) $$\delta t_{A}=\left|t_{A}^{\prime }-t_{A}\right|+1$$

Based on the above analysis, the antenna beam azimuth pointing measurement error is:

(12) $$\delta \alpha =\sqrt{{\left(\delta t_{A}\right)}^{2}+{\left(\delta t_{B}\right)}^{2}}\cdot V_{S}/R_{0}$$

where δα is the squint angle measurement error of the antenna beam, the unit is radians.

## 5. GaoFen-3 Test Data Processing Results

Gaofen-3 (GF-3) is China’s first meter-level multi-polarization synthetic aperture radar (SAR) satellite with scientific and commercial applications, which was developed by the China Academy of Space Technology and had been launched in August 2016 [25].

### 5.1. Test Information and Data

On September 8, 2016, in the Ordos microwave calibration field in Inner Mongolia, an external field calibration experiment was conducted and a polarized ground receiver was installed. For the H and V polarized pulse signals alternately emitted by the GF-3 SAR satellite, the relevant parameters of the test are as follows:

- PRF = 2792.176270 Hz;
- eqvPRF = 1396.088135 Hz;
- centerLookAngle = 29.360000°;
- azimuth emission pattern −3 dB beamwidth = 0.188°;
- Fs = 66.66667 MHz;
- Bandwidth = 60 MHz;
- pulsewidth = 24.99 us;
- satVelocity = 7567.397210 m/s;
- groundVelocity = 6738.85526 m/s;
- averageAltitude = 1228.350098 m;
- nearRange = 874,626.096672 m.

The location of the ground receiver is:

- Latitude = 39.2411° N;
- Longitude = 107.4662° E;
- Altitude = 1320.60 m.

The antenna pattern parameters of the ground receiver is:

- receiving pattern −3 dB beamwidth = 13.6°;
- receiving pattern −0.5 dB beamwidth = 5.6°.

The data of the ground receiver is:

- samplingrate = 300 MHz;
- RangeofSat2Receiver = 882,300.41 m.

**Figure 7:**
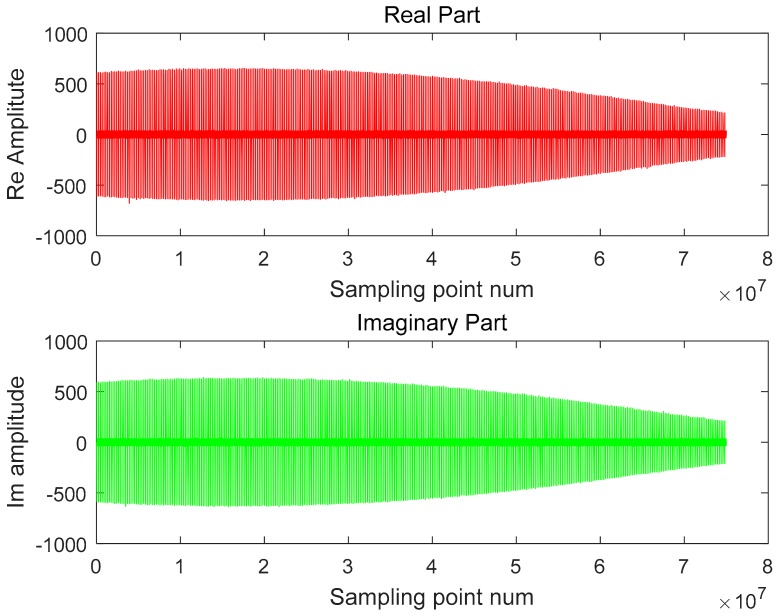
Complex sampling data recorded by the ground receiver.

The H-polarized data recorded by the ground receiver are shown in Figure 7 below. The sampling rate of the receiver is 300 MHz. The recorded number of complete H-polarized transmit pulses is 349 and the length of time is about 0.25 s. It can be seen from the figure that the main lobe portion of the SAR antenna beam is received. The SAR azimuth emission pattern beamwidth and the ground receiver receiving pattern beamwidth data show that the latter is more than 72 times that of the former, so the modulation effect of the receiver pattern can be ignored in the receiver data processing.

### 5.2. Test Data Processing and Analysis

**Figure 8:**
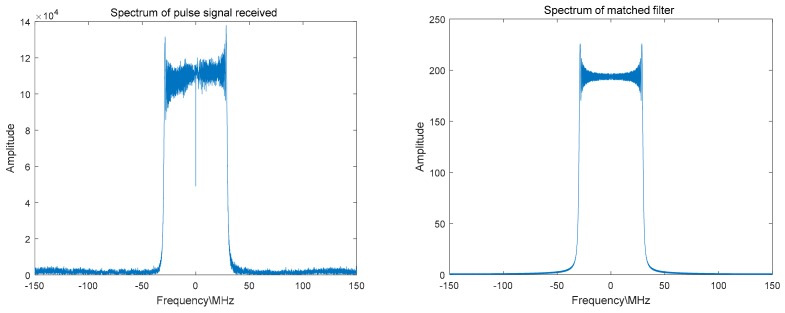
Spectrum of one of the pulse signals and the constructed matched filter.

**Figure 9:**
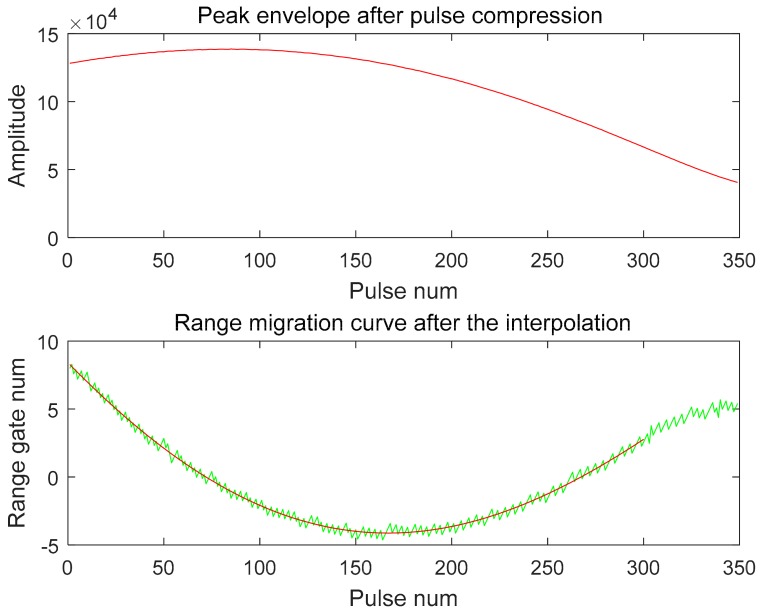
Extracted range migration curve and peak envelope after pulse compression.

In order to improve the processing efficiency, the pulse signal data is first taken out from the complex sampling data, and the invalid noise data between pulses is eliminated; then, process the data as described in Section 3. Figure 8 shows the spectrum of one of the pulse signals and the spectrum of the matched filter. Figure 9 shows the extracted range migration curve and the pulse compression peak envelope.

**Figure 10:**
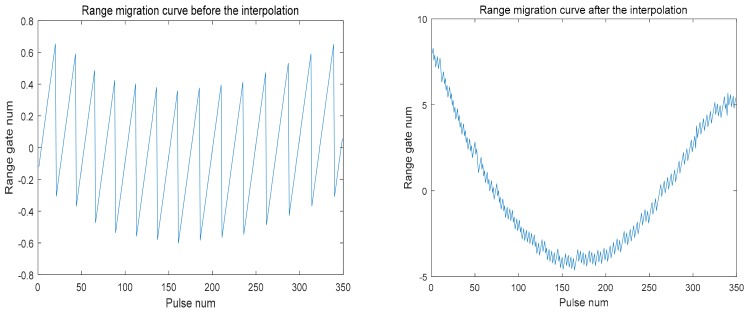
The comparison of the range migration curves before and after interpolation.

According to the pulse signal, acquisition time length is 0.25 s, and according to the satellite flight speed, the range from satellite to receiver and other parameters and supposing that the ground receiver collected the data of the peak time, the maximum possible range migration is calculated to be 2.02827 m. The one-way range resolution calculated from the above parameters is 5 m, and the sampling interval of the ground receiver corresponds to 1 m, so that the change of the entire range migration curve does not exceed 0.4 range gates and the corresponding sampling interval is 2 units. In this case, it is impossible to measure the extreme position at all. Figure 10 shows the comparison of the effects before and after interpolation. It can be seen that after 32 multiples interpolation, the range migration changes reach 12–13 units. There is also better symmetr so that the extreme point of the range migration curve can be accurately obtained.

**Figure 11:**
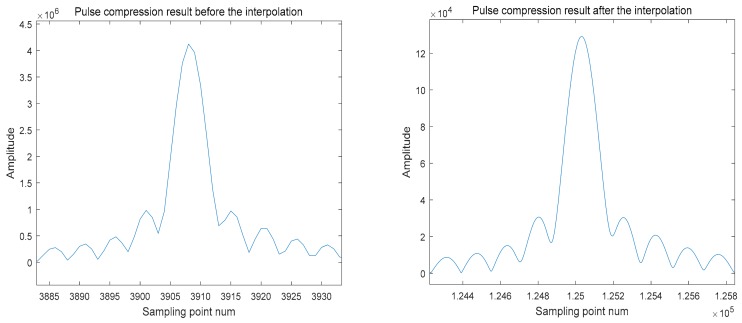
The comparison of the pulse compression results before and after interpolation.

Figure 11 shows the comparison of the pulse compression results before and after interpolation, and shows that only the peak value of the pulse compression can be accurately extracted after interpolation.

It can also be seen from Figure 9 that the pulse signal amplitude envelope center is also obtained and is not at the edge position, thus the method of Section 3 can also be used to solve the center position of the main lobe of the pattern. However, the M value cannot be obtained too large; this is the limitation of the data acquired by this receiver.

Using the method of Section 3, the center position of the measured main lobe of the antenna pattern is the 85th pulse in the measured data and the 86th pulse in the fitting curve of the measurement data. The extreme position of the range migration curve is the 164th pulse in the measured data and the 167th pulse in the fitting curve of the measurement data. According to the spatial relationship between the satellite and the ground receiver, the range between the satellite and the ground receiver is calculated as $R_{0}$ = 882,300.41 m. Calculating the antenna beam squint angle deviation according to formula (8) is:

Δα=0.0285°

According to Equation (12), the estimation accuracy is approximately:

δα=0.00157°

## 6. Conclusions

In this paper, a method for accurate measurement of spaceborne SAR’s antenna beams azimuth pointing based on ground receivers is proposed. This method is based on the fact that ground receivers can receive the complex sampling data of the pulse signals transmitted from the spaceborne SAR. These complex sampling data contain the information of the antenna pattern and the information of the changes in the positions of the satellite and receiver. These two kinds of information are in the same time coordinate, which provides the basis for high precision. The processing results of the GF-3 SAR test data show that the method effectively measures the azimuth direction of the antenna beam. For LEO satellites, the measurement accuracy reaches 0.002°, which is equivalent to TerraSAR-X’s method.

## Appendix A

When the three attitude angles of yaw, rolling and pitch angle are all 0, the satellite body coordinate system coincides with the orbit coordinate system. When the attitude angle is not zero, the orbit coordinate system $OX_{o}Y_{o}Z_{o}$ is converted to the satellite body coordinate system $OX_{b}Y_{b}Z_{b}$ by three coordinate rotations, and the conversion order is Z(ψ)−X(ϑ)−Y(θ), the transformation matrix $C_{orbit2star}$ can be expressed as:

(A1) $$C_{orbit2star}=\left[\begin{array}{ccc} \cos\theta & 0 & -\sin\theta \\ 0 & 1 & 0\\ \sin\theta & 0 & \cos\theta \end{array}\right]\left[\begin{array}{ccc} 1 & 0 & 0\\ 0 & \cos\vartheta & \sin\vartheta \\ 0 & -\sin\vartheta & \cos\vartheta \end{array}\right]\left[\begin{array}{ccc} \cos\psi & \sin\psi & 0\\ -\sin\psi & \cos\psi & 0\\ 0 & 0 & 1 \end{array}\right]$$

where ψ is the yaw attitude angle, ϑ is the rolling attitude angle, and θ is the pitch attitude angle.

The angular relationship of the antenna coordinate system $O_{a}X_{a}Y_{a}Z_{a}$ with respect to the satellite body coordinate system $OX_{b}Y_{b}Z_{b}$ is given by stand-alone precision measurement (or theoretical design value). The Table A1 below gives the angle measurement of the three coordinate axes of the antenna coordinate system and the three coordinate axes of the satellite body coordinate system.

**Table A1:** Antenna coordinate system stand-alone angle fine measurement data.

	*OX_b_Y_b_Z_b_*	*X_b_*	*Y_b_*	*Z_b_*
*O_a_X_a_Y_a_Z_a_*				
*X_a_*		$\theta_{1}$	$\theta_{2}$	$\theta_{3}$
*Y_a_*		$\theta_{4}$	$\theta_{5}$	$\theta_{6}$
*Z_a_*		$\theta_{7}$	$\theta_{8}$	$\theta_{9}$

According to the precision measurement result, the conversion matrix $C_{star2antenna}$ of the satellite body coordinate system $OX_{b}Y_{b}Z_{b}$ to the antenna coordinate system $O_{a}X_{a}Y_{a}Z_{a}$ is:

(A2) $$C_{star2antenna}=\left[\begin{array}{ccc} \cos\theta_{1} & \cos\theta_{2} & \cos\theta_{3}\\ \cos\theta_{4} & \cos\theta_{5} & \cos\theta_{6}\\ \cos\theta_{7} & \cos\theta_{8} & \cos\theta_{9} \end{array}\right]$$
